# A characteristic MRI finding to diagnose a partial tear of the medial meniscus posterior root: an ocarina sign

**DOI:** 10.1186/s43019-021-00120-4

**Published:** 2021-10-09

**Authors:** Takayuki Furumatsu, Takaaki Hiranaka, Keisuke Kintaka, Yuki Okazaki, Naohiro Higashihara, Masanori Tamura, Toshifumi Ozaki

**Affiliations:** Department of Orthopaedic Surgery, Okayama University Hospital, 2-5-1 Shikatacho, Kitaku, Okayama 700-8558 Japan

**Keywords:** Medial meniscus, Posterior root, Partial tear, Magnetic resonance imaging, Ocarina sign

## Abstract

**Background:**

Diagnosing partial tears of the medial meniscus (MM) posterior root is difficult. The aim of this study was to evaluate diagnostic values involved in conventional magnetic resonance imaging (MRI) features of MM posterior root tears (MMPRTs) and find other MRI-based findings in patients with partial MMPRTs.

**Methods:**

Eighteen patients who had arthroscopically confirmed partial MMPRTs were included. As a control, 18 patients who underwent partial meniscectomy for other types of MM tears were evaluated. Isolated partial MMPRTs were classified into the following three types: type A, accurate partial stable tear (cleavage < 1/2 of root width); type B, bridged unstable root tear (cleavage ≥ 1/2 of root width); type C, complex horn tear expanded to the root. Conventional MRI-based findings of MMPRTs were evaluated between two groups (*n* = 23). Posterior root irregularity, bone marrow spot, and ocarina-like appearance showing several condensed circles in triangular meniscal horn (ocarina sign) were also evaluated.

**Results:**

Posterior root irregularity and bone marrow spot were frequently observed in the partial MMPRTs (47.8%), compared with the other MM tears (*P* = 0.007 and 0.023, respectively). The ocarina sign was detected in 69.6% of patients with partial MMPRTs. A significant difference between two groups was observed in a positive ratio of ocarina sign (*P* < 0.001). Types A, B, and C of the partial tear/damage were observed in three, eight, and seven patients, respectively. The ocarina sign was the most common MRI finding in each type of partial MMPRT.

**Conclusions:**

This study demonstrated that a characteristic MRI finding, “ocarina sign,” was frequently observed in patients with partial tear/damage of the MM posterior root. The ocarina sign was the most common MRI finding in several types of partial MMPRTs. Our results suggest that the ocarina sign may be useful to diagnose unnoticed partial MMPRTs.

*Level of evidence:* IV, retrospective comparative study.

## Background

Posterior root tears of the medial meniscus (MM), including partial, complete radial, and/or oblique tears adjacent to the posterior root attachment, lead to progressive cartilage loss, osteoarthritis, and subchondral insufficiency fracture of the knee joint by disrupting the MM functions [1]. With a greater understanding of post-traumatic knee degradation following MM posterior root tear (MMPRT), more emphasis has been placed on accurate diagnosis and early identification of MMPRT by magnetic resonance imaging (MRI) examinations [2, 3]. Several characteristic MRI findings have been reported to detect MMPRT. In coronal images, giraffe neck sign and cleft/truncation sign (vertical linear defect) are useful to diagnose MMPRT [4–8]. In sagittal images, a ghost (or white meniscus) sign that shows a disappearance of the MM posterior root/horn on some slices can diagnose MMPRT with high sensitivity. A radial tear sign (radial linear defect) with complete discontinuity of the posterior root and fluid gap on axial images also has high diagnostic value in the diagnosis of MMPRT [4–6]. On the other hand, MM medial extrusion sign is often observed in the other types of MM tears and has a lower accuracy for detecting MMPRT compared with giraffe neck, cleft, ghost, and radial tear signs [5, 6]. However, these signs are clinically useful to detect complete tears of the MM posterior root. Diagnosing partial tear and/or pathological damage of the MM posterior root is still difficult.

A partial tear of the MM posterior root sometimes progresses to a complete radial root tear of the MM during nonoperative management [9]. Choi et al. report the MMPRTs as posterior MM root ligament lesions, and these lesions include three types of MRI-based appearances: degeneration, characterized by thickening of the root with intrasubstance hyperintensity not contacting the articular surface; partial tear, characterized by abnormal signal intensity extending to the articular surface or abnormal morphology of the root with partial root discontinuity; and complete tear, characterized by complete discontinuity of the affected root [10]. Palisch et al. describe that a partial radial tear of the MM posterior root (LaPrade arthroscopic/morphologic classification type 1 [11]) shows a fluid signal intensity at the root insertion and the subchondral and/or subenthesial linear bone marrow signal intensity on MRI [2]. However, the MM posterior root usually consists of multiple fiber bundles, typically exhibited as hyperintensity-/hypointensity-mixed signals (most of which are stripe-like signals) on MRI [12]. Many other meniscus tears at the MM posterior horn (horizontal, radial, and/or complex tears) show subcortical cystic lesions and/or posterior shiny-corner lesions (bone marrow lesions at the meniscal-covered portions of the tibial plateau) around the MM posterior root insertion [13, 14]. In addition, these studies have not proven congruity between MRI-based findings and actual arthroscopic views of meniscus tears.

The aim of this study was to evaluate diagnostic values involved in conventional MRI features of the MMPRT and find other MRI-based findings in patients with arthroscopically confirmed partial tears of the MM posterior root. We hypothesized that specific degenerative findings at the MM posterior root/horn on MRI are clinically useful to identify partial tear/damage of the MM posterior root.

## Patients and methods

This study received the approval of our institutional review board, and written informed consent was obtained from all patients. Eighteen patients who underwent transtibial pullout repairs for arthroscopically confirmed partial tears of the MM posterior root between February 2018 and December 2020 were included (Table 1). As a control group, 18 patients who underwent partial meniscectomy for other types of MM tears between July 2018 and November 2020 were evaluated (Table 1). All the patients required arthroscopic treatments for symptomatic knee pains and were diagnosed as having isolated MM tears by MRI examinations and arthroscopic findings [15]. Details of sudden posteromedial painful popping, a characteristic episode of patients with MMPRTs, were obtained from the patients by careful interviews [16, 17]. Patient demographics are presented in Table 1.

**Table 1:** Patient demographics

	Partial MMPRTs	Other MM tears	*P* value
Number of patients	18	18	
Gender, men/women	2/16	11/7	0.005*^a^
Age (years)	64.6 ± 7.8	54.0 ± 9.0	0.001*
Height (m)	1.54 ± 0.06	1.66 ± 0.09	< 0.001*
Body weight (kg)	62.3 ± 11.2	69.4 ± 13.5	0.046*
Body mass index (kg/m^2^)	26.2 ± 3.9	25.1 ± 3.7	0.184
Painful popping episode (%)	8 (44.4)	2 (11.1)	0.060^a^
MRI examinations	23	23	

Data of age, height, body weight, and body mass index are displayed as mean ± standard deviation. *MMPRT* medial meniscus posterior root tear, *MM* medial meniscus. Statistical differences in age, height, body weight, and body mass index between two groups were analyzed using Mann–Whitney *U* test. ^a^Fisher’s exact test. *Significant difference

### Arthroscopic evaluations of the MM tears

Following the medial joint space-widening procedure (outside-in pie-crusting technique [18]), partial MMPRTs were determined by careful arthroscopic examinations according to the meniscal root tear classification [11]. A partial tear/damage of the MM posterior root was defined as an incomplete structural cleavage between 0 and 9 mm from the native MM posterior root attachment. An “accurate” partial stable tear (type A) of the MM posterior root was determined by careful observation with probing. A structural cleavage with the root width of less than one half was classified as a type A partial tear. A superficially “bridged” root covered with connective tissues was classified as a type B partial tear that showed a structural cleavage with root width of one half or more. A “complex” tear of the MM posterior horn expanded to the posterior root was classified as a type C partial tear. A completely detached root with a gap was excluded in this study. Other types of MM tears were determined by arthroscopic findings and MRI scans: ten complex tears involved in degenerated horizontal/flap tears, four flap tears, three degenerated horizontal tears, and one degenerated bucket-handle tear.

A partial tear/damage of the MM posterior root was divided into the following three types: type A, accurate partial stable tear (cleavage < 1/2 of root width); type B, bridged unstable root tear (cleavage ≥ 1/2 of root width); type C, complex horn tear expanded to the root.

### Assessments of MRI scans

Retrospective re-evaluations of preoperative MRIs were performed after the surgeries. Patients were examined by preoperative MRI scans (one to three times). The number of MRI examinations was 23 for each group (partial MMPRTs or other MM tears). MRI scans were mainly obtained using an Achieva 1.5T (Philips, Amsterdam, The Netherlands) or an EXCELART Vantage Powered by Atlas 1.5T (Toshiba Medical Systems, Otawara, Japan) with a knee coil. Standard sequences of the Achieva included sagittal [repetition time (TR)/echo time (TE) 742/18], coronal (TR/TE 637/18), and axial (TR/TE 499/18) T2-weighted fast-field echo with a 20° flip angle (FA). Standard sequences of the Vantage included sagittal and coronal proton density (PD) fast-spin-echo (TR/TE 2300/18) and axial T2-weighted fat suppression (TR/TE 3500/60) with a 90° FA. Slice thickness was 3 mm with a 0.6 mm gap. Field of view (FOV) was 16 (or 17) cm with an acquisition matrix size of 205 × 256 (or 200 × 368) [6, 19]. Coronal images were obtained, along with a section parallel to a tangential line between both posterior femoral condyles. Sagittal images were set perpendicular to the coronal images. Axial images were obtained according to the position of both menisci.

Conventional MRI-based findings of the MMPRT such as cleft, giraffe neck, medial extrusion, ghost, and radial tear signs [6] were evaluated. Medial extrusion of the MM was measured from the medial margin of the tibial plateau to the outer border of the MM on the coronal image that crossed the midpoint of the anteroposterior length of the MM. Medial extrusion (> 3 mm) was defined as a positive extrusion sign.

**Fig. 1:**
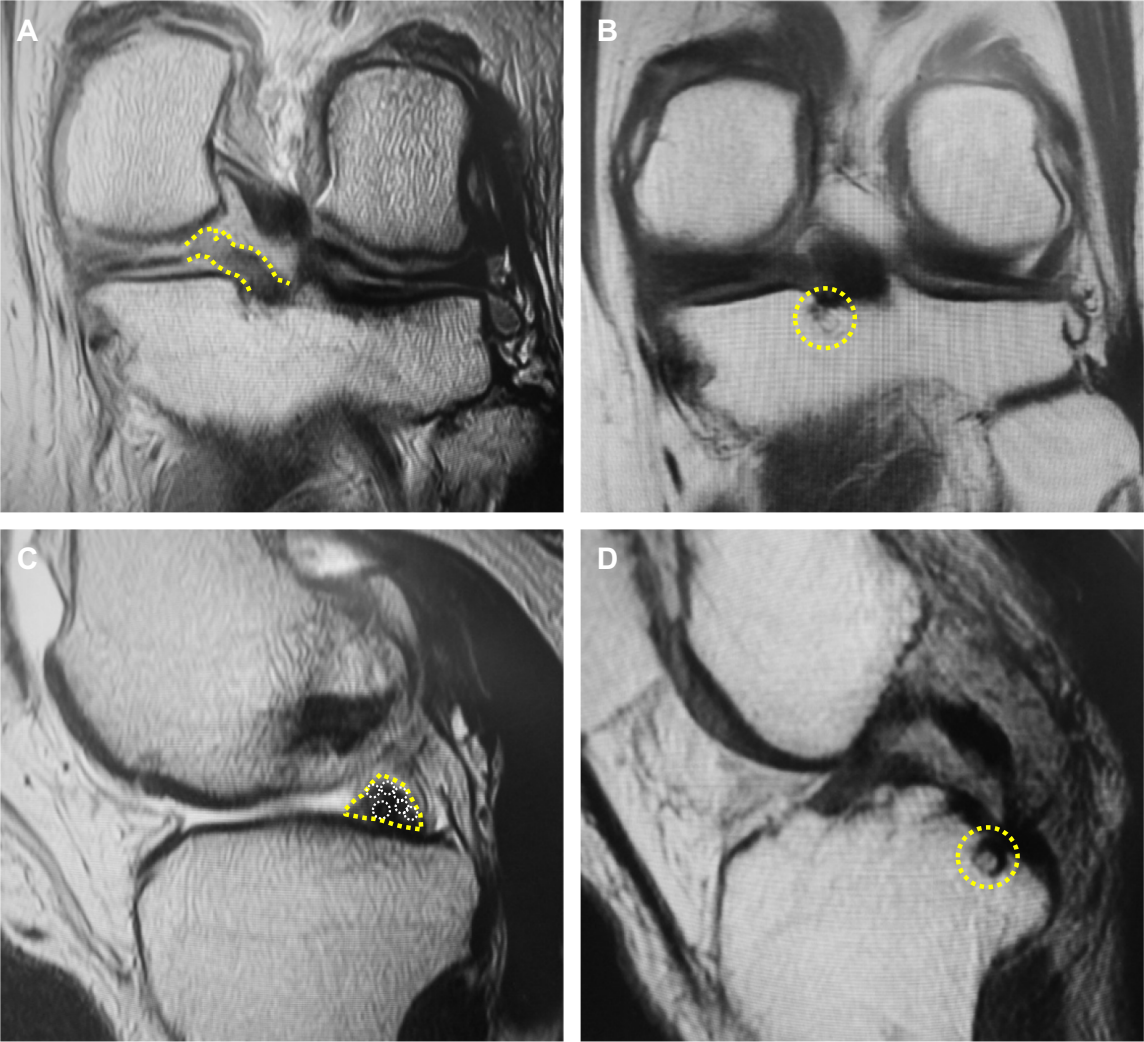
Characteristic MRI finding in partial tear/damage of the MM posterior root. **A** A root irregularity (dotted area, coronal view). **B** A bone marrow spot (subcortical cyst) under the posterior root attachment (dotted circle, coronal view). **C** An ocarina sign (dotted area, sagittal view nearby the MM posterior root). **D** A bone marrow spot (dotted circle, sagittal view)

**Fig. 2:**
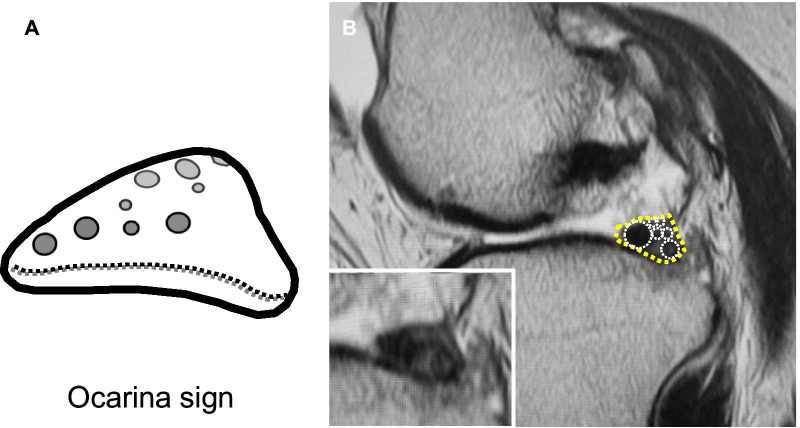
An ocarina sign. **A** An illustration of an ocarina sign. **B** A sagittal image of a partial MMPRT. Note a multiple fiber bundle formation showing several condensed circles (white circles) in triangular meniscal horn (yellow dotted area), an ocarina-like appearance. An inlet shows the original image

During MRI evaluations, we noticed that root irregularity and bone marrow spot at the posterior root attachment were frequently observed in coronal/sagittal images of the patients (Fig. 1). A bone marrow spot (subcortical cyst) was defined as foci of markedly increased signal intensity in the subcortical bone with rounded and well-circumscribed margins and no evidence of internal marrow tissue or trabecular bone on both the T2 and PD-weighted images [13]. In sagittal images, a multiple fiber bundle formation showing several condensed circles in triangular meniscal horn, an ocarina-like appearance, was observed with considerable frequency (Figs. 1, 2). Root irregularity, bone marrow spot, and “ocarina sign” were evaluated as characteristic MRI findings to identify partial tear/damage of the MM posterior root.

### Statistical analysis

Data were presented as means ± standard deviations. Statistical differences in age, height, body weight, body mass index, and MM extrusion between two groups were analyzed using Mann–Whitney *U*-test. Differences in gender, positive ratio of painful popping episode, and MRI signs between groups were compared using Fisher’s exact test. Power and statistical analyses were performed using EZR (Saitama Medical Center, Saitama, Japan), which is a graphical user interface for R (The R Foundation for Statistical Computing). Significance was set to *P* < 0.05. Two orthopedic surgeons independently assessed MRIs in a blinded manner. Each observer performed each evaluation twice, at least 2 weeks apart. The reliability of MRI evaluation was assessed by examining the interobserver and intraobserver reliabilities. The interobserver and intraobserver reliabilities were assessed using an intraclass correlation coefficient (ICC). ICC > 0.80 was considered to represent a reliable measurement.

## Results

The incidence of the MMPRT was higher in women compared with that of other MM tears (*P* = 0.005, Table 1). Significant differences between the MMPRT and other MM tear groups were observed in age (*P* = 0.001), height (*P* < 0.001), and body weight (*P* = 0.046). Posteromedial painful popping of the knee was not a significant characteristic episode in patients with partial MMPRTs (44.4%) compared with other MM tears (11.1%).

Conventional MRI findings of MMPRTs were evaluated in both groups (Table 2). A giraffe neck sign was observed in 21.8% of the MRIs with partial MMPRTs (*P* = 0.049). On the other hand, no significant differences between two groups were detected in cleft sign, MM extrusion, ghost sign, and radial tear sign (Table 2).

**Table 2:** Characteristic MRI findings in partial/complete MMPRTs

	Partial MMPRTs	Other MM tears	*P* value
Coronal images			
Cleft sign (%)	4 (17.4)	0 (0.0)	0.109^a^
Giraffe neck sign (%)	5 (21.8)	0 (0.0)	0.049*^a^
MM extrusion (mm)	3.21 ± 1.07	2.84 ± 1.21	0.142^b^
MM extrusion > 3 mm (%)	13 (56.5)	12 (52.2)	1.000^a^
Root irregularity (%)	11 (47.8)	2 (8.70)	0.007*^a^
Bone marrow spot (%)	11 (47.8)	3 (13.0)	0.023*^a^
Sagittal images			
Ghost sign (%)	1 (4.3)	0 (0.0)	1.000^a^
Ocarina sign (%)	16 (69.6)	3 (13.0)	< 0.001*^a^
Bone marrow spot (%)	8 (34.8)	2 (8.70)	0.071^a^
Axial images			
Radial tear sign (%)	3 (13.0)	0 (0.0)	0.233^a^

Data of medial meniscus (MM) extrusion are displayed as mean ± standard deviation. *MRI* magnetic resonance imaging, *MMPRT* medial meniscus posterior root tear. ^a^Fisher’s exact test. ^b^Mann–Whitney *U* test. *Significant difference

Posterior root irregularity and bone marrow spot (subcortical cyst) on coronal images were frequently observed in the partial MMPRTs (47.8%), with significant differences compared with other MM tears (*P* = 0.007 and 0.023, respectively). In sagittal images, a multiple fiber bundle formation that showed an ocarina-like appearance in triangular MM posterior horn (ocarina sign) was observed on 69.6% of MRI scans of the partial MMPRT group (Table 2, Figs. 1, 2). A significant difference between two groups was detected in a positive ratio of ocarina sign (*P* < 0.001). No significant difference between the groups was observed in bone marrow spot sign on sagittal images (*P* = 0.071).

**Fig. 3:**
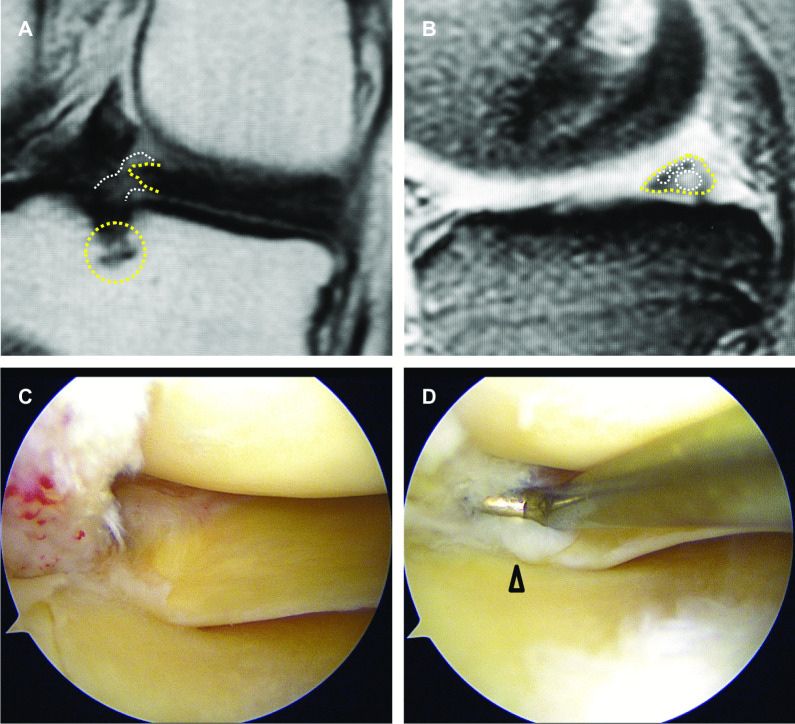
An “accurate” partial stable tear (type A) of the MM posterior root (64-year-old woman). **A** A posterior root irregularity (dashed area, PD-weighted coronal view). Bone marrow spots (yellow circle). **B** An ocarina sign (T2* sagittal view). **C**, **D** Arthroscopic findings from the anterolateral portal. A structural cleavage with root width > 1/2 (arrowhead)

**Fig. 4:**
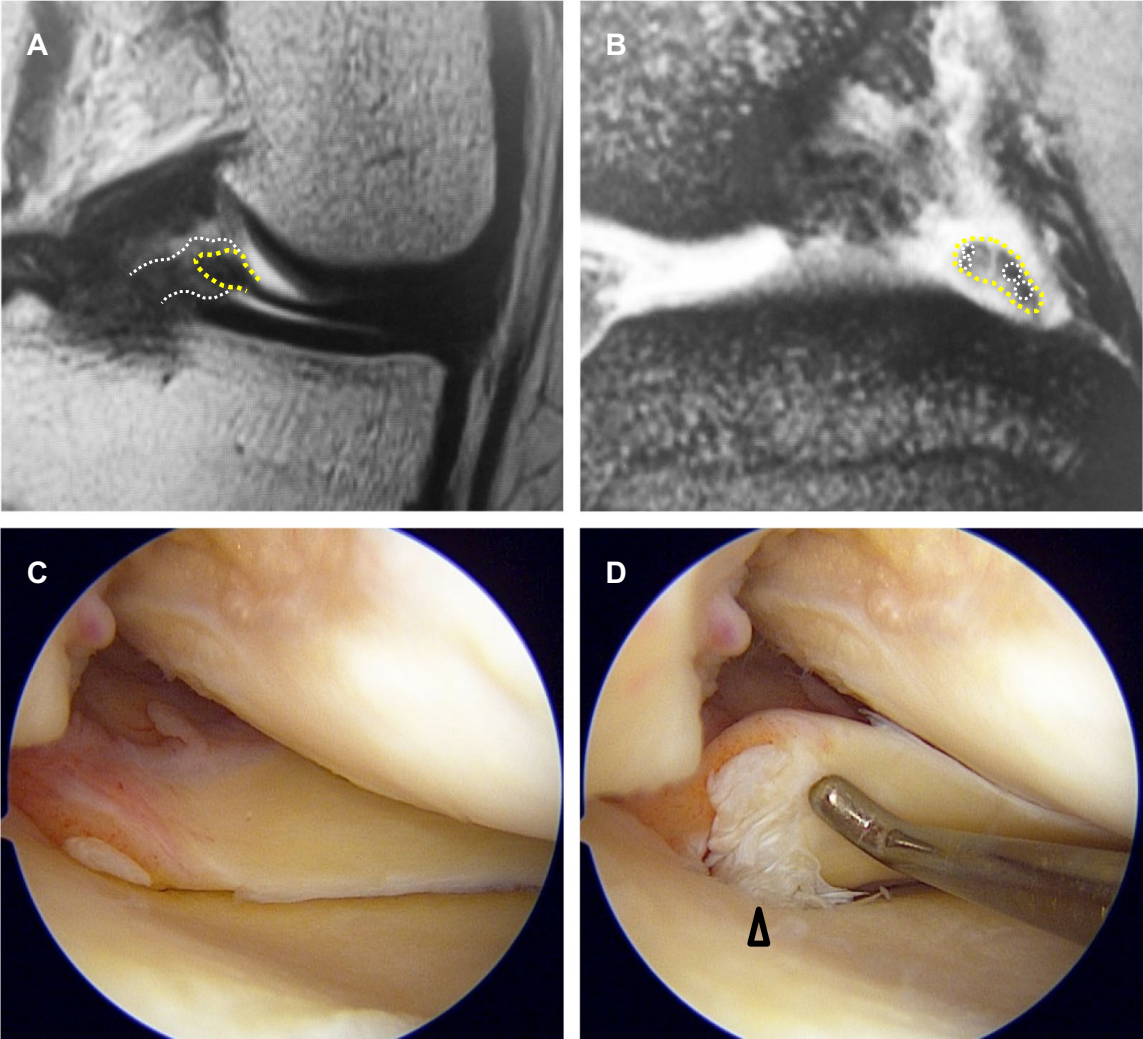
A “bridged” unstable root tear (type B) of the MM posterior root (61-year-old man). **A** A posterior root irregularity (dashed area, coronal view). **B** An ocarina sign (T2* sagittal view). **C**, **D** Arthroscopic findings from the anterolateral portal. An unstable tear (≥ 1/2 of root width) with connective tissue coverage (arrowhead)

**Fig. 5:**
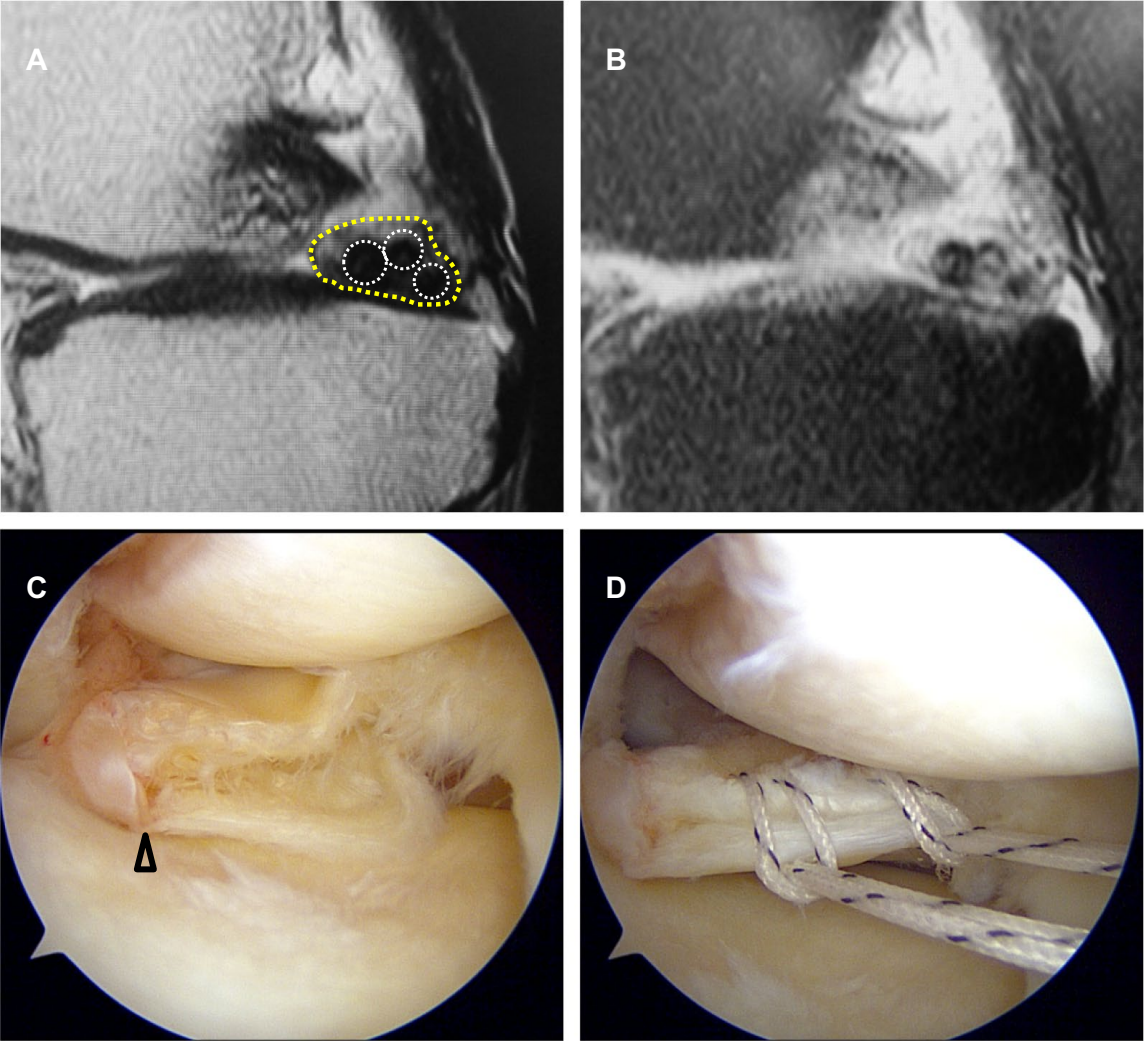
A “complex” tear of the MM posterior horn expanded to the MM posterior root (type C, 58-year-old woman). **A** An ocarina sign (sagittal view). **B** PD fat-saturated sagittal view. **C** Arthroscopic findings from the anterolateral portal. A complex posterior horn tear expanded to the root (arrowhead). **D** Two cinch stitches for posterior root repair

Accurate partial stable tears (cleavage < 1/2 of root width) of the MM posterior root were observed in three patients (type A, Fig. 3). Bridged unstable root tears (cleavage ≥ 1/2 of root width, type B, Fig. 4) and complex posterior horn tears expanded to the root (type C, Fig. 5) were observed in eight and seven patients, respectively. These three types of partial MMPRTs showed some continuity between the posterior root and horn in both arthroscopic and MRI findings. Ocarina sign was the most common MRI finding in each type of partial MMPRT (Table 3). There were no individual features of ocarina sign in each type of partial MMPRT. The interobserver reproducibility and intraobserver repeatability of the MRI findings were satisfactory, with mean ICC values of 0.87 and 0.89, respectively.

**Table 3:** Characteristic MRI findings in each type of partial MMPRTs

	Type A (*n* = 3)	Type B (*n* = 8)	Type C (*n* = 7)
Coronal images			
Cleft sign	0	3	0
Giraffe neck sign	0	3	1
Root irregularity	0	6	3
Bone marrow spot	3	3	3
Sagittal images			
Ghost sign	0	1	0
Ocarina sign (%)	2 (67)	6 (75)	5 (71)
Bone marrow spot	2	3	2
Axial images			
Radial tear sign	0	2	1

Evaluations of most recent magnetic resonance imaging (MRI) before surgery. *MMPRT* medial meniscus posterior root tear

## Discussion

The most important finding in this study was that a new characteristic MRI finding, “ocarina sign,” was frequently observed in patients with partial tear/damage of the MM posterior root. In addition, the ocarina sign was the most common MRI finding in several types of partial MMPRTs. Our results suggest that the ocarina sign may be a useful MRI finding to diagnose an unnoticed partial tear/damage of the MM posterior root.

Choi et al. describe that partial tears of the MM posterior root were observed in 11.7% of 419 symptomatic knee MRIs [10]. Degenerations and complete tears of the MM posterior root were detected in 14.3% and 2.6% of MRI scans in patients with symptomatic knees, respectively. In this literature, the rate of partial tears account for 40.8% (49/120) of MRI-based posterior MM root ligament lesions, despite a lower rate of complete tears (9.2%, 11/120) [10]. On the other hand, Moon et al. describe that three (4.8%) patients showed partial MMPRTs corresponding to LaPrade classification type 1 tears in arthroscopic image analyses of 63 patients [20]. They report that complete radial (type 2) and complex oblique (type 4) tears accounted for 88.9% and 6.3% of the MMPRTs, respectively [20]. Several authors demonstrate that the rate of type 1 partial MMPRTs accounts for 3.9% (5/128 knees) [21], 7.2% (6/83 knees) [22], and 16.4% (19/116 knees) [23] in patients with MMPRTs who underwent arthroscopic treatments. Based on these findings, most of the patients diagnosed with MRI-based partial MMPRTs may not require immediate surgical treatments. Many of the partial tear/damage of the MM posterior root may not be noticed on clinical examination and MRI assessment, even if a surgeon request MRI scans. We consider that several useful MRI findings such as ocarina sign, root irregularity, and bone marrow spot can help surgeons and radiologists to make a reliable diagnosis of partial MMPRTs for preventing further progression to complete MMPRTs.

This study demonstrated that the incidence of the MMPRT was higher in women compared with that of other types of MM tears (Table 1). In addition, the MMPRTs were detected in older patients (mean age, 64.6 ± 7.8 years) compared with other MM tears (54.0 ± 9.0 years). Several authors report that MMPRTs can occur particularly in middle-aged or older female patients who have a sudden posteromedial painful popping sensation of the knee during light activities such as using stairs and walking [5, 16, 17]. The posteromedial painful popping sensation is a useful clinical symptom to identify MMPRTs, and its sensitivity is 35–87% in patients with MMPRTs [16, 24]. However, in our study, no significant difference between partial MMPRT and other MM tear groups was observed in the positive ratio of painful popping episode (Table 1). A relatively low rate (44%) of the painful popping episode may be caused by the presence of type C complex posterior horn tear (Fig. 5) that expands to the MM posterior root gradually. On the other hand, five out of eight patients (63%) who had type B bridged unstable root tears, similar to complete radial MMPRTs, remembered the popping episode.

In human cadaveric knees, fibrocartilage metaplasia and calcification increase in the MM posterior roots is associated with the degree of the MMPRT (partial or complete tears). A decrease of type I collagen deposition and an increase of type II collagen synthesis is observed in the extracellular matrix of the MM posterior root in osteoarthritic knees [25]. In addition, meniscus cell clusters that show depositions of type II collagen, aggrecan, and safranin O-stained proteoglycans are frequently observed around meniscus tears [26]. These findings suggest that age-dependent degenerative changes in the MM posterior root may lead to partial and/or complete MMPRTs in older patients. We consider that the ocarina sign may represent some fiber-bundle disorganization and fibrocartilage formation around the junction between the MM posterior root and horn. Further investigations using histological analyses will be required to understand the ocarina-like appearance in MRI scans. An anatomic attachment of the MM posterior root is surrounded with the lateral edge of the medial tibial plateau, the anterior border of the posterior cruciate ligament, and the retro-eminence ridge. The maximum anteroposterior length of the MM posterior root attachment is a mean of 7.7 mm at the sagittal plane passing through the peak of the medial intercondylar tubercle [27]. The mediolateral width of the MM posterior root attachment is shorter than its anteroposterior length [27, 28]. In our study, the difference in the detection rate of bone marrow spot under the MM posterior root attachment was observed between coronal and sagittal images on MRI scans (Table 2). This may be caused by the difference in the number of MRI sections passing through the anatomic attachment of the MM posterior root.

There are several limitations in this study. Our study was a retrospective comparative study that included a small number of patients. MRI examinations were performed differently (number of examination times, variable protocols, and duration from injury to MRI scans). There is a possibility that the appearance of the ocarina sign may depend on the duration between injury and MRI examination. We evaluated several characteristic MRI findings in a single knee flexion angle (10°) under non-weight-bearing condition. MRI assessments of the MM posterior root attachment using thin slices and appropriate planes under loading condition may enhance the diagnostic value of these characteristic MRI findings in identifying partial tear/damage of the MM posterior root.

## Conclusions

This study demonstrated that a characteristic MRI finding, the “ocarina sign,” was frequently observed in patients with partial tear/damage of the MM posterior root. In addition, the ocarina sign was the most common MRI finding in several types of partial MMPRTs. Our results suggest that the ocarina sign, root irregularity, and bone marrow spot under the root attachment may be useful MRI findings to diagnose unnoticed partial tears/damage of the MM posterior root.

## Data Availability

Not applicable.

## Abbreviations

MM: Medial meniscus
MMPRT: Medial meniscus posterior root tear
MRI: Magnetic resonance imaging
